# The versatility of the putative transient receptor potential ion channels in regulating the calcium signaling in *Aspergillus nidulans*

**DOI:** 10.1128/msphere.00549-23

**Published:** 2023-11-16

**Authors:** Hongchen Wang, Renwei Gao, Yuanwei Zhang, Ling Lu

**Affiliations:** 1 Jiangsu Key Laboratory for Microbes and Functional Genomics, Jiangsu Engineering and Technology Research Center for Microbiology, College of Life Sciences, Nanjing Normal University, Nanjing, China; CNRS-INSERM-Université Côte d'Azur, Nice, France

**Keywords:** transient receptor potential, *Aspergillus nidulans*, calcium channel

## Abstract

Transient receptor potential (TRP) channels are evolutionarily conserved integral membrane proteins. They serve as crucial mediators of sensory signals and have profound effects on cellular functions. However, many functions of the TRP family remain to be explored. In this study, the biological functions of three potential TRP channels, TrpA, TrpB, and TrpC, in *Aspergillus nidulans* have been verified. Green fluorescent protein labeling indicated that these TRP channels have distinct localization patterns. TrpA was primarily located at hyphal tips, TrpB showed a localization pattern resembling the Golgi apparatus, and TrpC displayed a possible ER-like localization pattern. Findings suggested that these TRP channels may be involved in different physiological processes. Deletion or reduced expression of TrpA resulted in severe growth defects and abolished conidiation. Moreover, TrpA may have multiple roles during fungal development, as TrpA mutants caused significant changes in gene expression. The lack of TrpB resulted in colony defects in a calcium-dependent manner, especially under high-temperature conditions. These defects in Δ*trpB* can be largely rescued by adding calcium, indicating that TrpB is required in low-calcium conditions. The deletion of TrpC did not exhibit any detectable phenotypes under tested cultural conditions. However, when major calcium channels MidA/CchA or PmrA were absent, the lack of TrpC further exacerbated the colony defects, implying that TrpC serves as a supplementary function. In conclusion, TRP channels are involved in a wide range of physiological processes and findings provide a valuable reference for investigating the functions of TRP proteins in other fungal or eukaryotic species.

**IMPORTANCE:**

Transient receptor potential (TRP) ion channels are evolutionarily conserved integral membrane proteins with non-selective ion permeability, and they are widely distributed in mammals and single-cell yeast and serve as crucial mediators of sensory signals. However, the relevant information concerning TRP channels in *Aspergillus nidulans* remains inadequately understood. In this study, by gene deletion, green fluorescent protein tagging, and cytosolic Ca^2+^ transient monitoring techniques, the biological functions of three potential TRP channels (TrpA, TrpB, and TrpC) have been explored for which they play distinct and multiple roles in hyphal growth, conidiation, responsiveness to external stress, and regulation of intracellular Ca^2+^ homeostasis. The findings of this study on the functions of potential TRP channels in *A. nidulans* may serve as a valuable reference for understanding the roles of TRP homologs in industrial or medical strains of *Aspergillus*, as well as in other filamentous fungi.

## INTRODUCTION

Transient receptor potential (TRP) proteins are non-selective and permeable ion channels that regulate a variety of processes in mammalian cells through the regulation of ion transport (1–4). Initially described in *Drosophila*, similar proteins with different ion permeability and gating mechanisms have subsequently been identified in most eukaryotes (3, 5–8). Presently, a total of 28 mammalian TRP members have been reported and categorized into six primary subfamilies based on their sequence homology: TRPC, TRPA, TRPM, TRPP, TRPV, and TRPML (6). Furthermore, the TRPN subfamily is expressed in invertebrates, such as flies and worms, along with cold-blooded vertebrates (9, 10). The TRPY subfamily, on the other hand, is expressed in yeasts (11). TRP proteins are primarily acknowledged for their roles in sensing environmental stimuli, which can elicit somatosensory sensations such as pain, cold, itching, and other defensive reactions (3, 12). Additionally, mutations in TRP proteins have been found to be associated with human diseases, indicating the significant physiological importance of TRP proteins (2, 7). The majority of mammalian TRP channels are Ca^2+^-permeable and function by assembling into a tetramer, with each polypeptide composed of six transmembrane domains (TMDs), and the putative ion-conducting pore situated between the fifth and sixth TMDs (2, 6, 13). These structure characterizations of intricate arrangement in TRP channels enable them to regulate ion transport and modulate various intracellular processes effectively (1). In addition to the TRP channel protein Yvc1p, several other TRP-like proteins have been identified in fungi (14–16). The TRP domain is considered as a distinctive feature of the TRP channel and is present in fungal proteins. In *Candida albicans* and *Saccharomyces cerevisiae*, TRP domain-containing proteins have been identified as heme uptake-related genes and categorized as members of the flavin carrier (FLC) family (17). This FLC family is responsible for transporting flavin adenine dinucleotide into the lumen of the endoplasmic reticulum (ER). However, these FLC proteins also play important roles in calcium homeostasis, cell growth regulation, maintenance of cell wall integrity, and response to osmotic shock (18). In *Schizosaccharomyces pombe*, the TRP-like ion channel Pkd2 has also been observed to function as a Ca^2+^-permeable channel and mediates the cytoplasmic Ca^2+^ response (19). Moreover, it has also been found to be involved in cell wall synthesis and the trafficking of membrane proteins (20–22). In subsequent studies, the functionalities of these TRP domain-containing proteins have been consistently investigated in other fungal species. It has been found that these proteins not only possess Ca^2+^ regulatory functions but also assume distinctive and significant roles in their respective species (22–25). In the opportunistic yeast pathogen *Cryptococcus neoformans*, Flc1, a homolog of FLC family proteins found in the genome of *S. cerevisiae*, has been postulated to be essential for regulating stress response and virulence (26, 27). Additionally, the deficiency of Flc1 in *C. neoformans* leads to an abnormal distribution of chitin, the inhibition of vacuole fusion, and the disruption of autophagy (26, 27). In the filamentous fungal pathogen *Aspergillus fumigatus*, the Flc proteins (FlcA, FlcB, and FlcC) have been shown their criticality in growth, calcium and iron homeostasis, and, most importantly, in the virulence of this pathogen (28, 29). Hence, we conclude that this potential class of TRP proteins holds considerable importance and exhibits functional diversity in fungi.

*Aspergillus nidulans* stands as a well-established model for the study of filamentous fungi. Due to its advanced genetic operating system and its capacity for multiple gene editing through easy operated crossing strategy between strains, *A. nidulans* has been extensively utilized as a research model to investigate the processes that govern the morphological development of fungi (30). Previous studies have unveiled that numerous genetic traits observed in *A. nidulans* can be extrapolated to other fungi and even eukaryotes (31). In *A. nidulans*, studies have reported that several pathways are involved in regulating fungal adaptability and response to various stresses (32–35). However, the relevant information concerning TRP channels remains inadequately understood. Using bioinformatics analysis of the *A. nidulans* genome, we have successfully identified four TRP-like proteins: AN9146 (*trpR*), AN1950 (*trpA*), AN7443 (*trpB*), and AN5367 (*trpC*), using the TRP Pfam (PF06011). We previously demonstrated that TrpR is a Golgi-localized TRP-like calcium channel that plays a crucial role in conidiation and cell wall integration. It is also involved in responding to thermal and cell wall stress by regulating calcium homeostasis in *A. nidulans* (36). In this study, we focused on the functions of three unknown TRP proteins, namely, TrpA, TrpB, and TrpC, and identified their roles in hyphal growth, conidiation, responsiveness to external stress, and regulation of intracellular Ca^2+^ homeostasis in *A. nidulans*.

## RESULTS

### Phylogenetic analysis of the candidate TRP channels in *A. nidulans*

**Fig 1:**
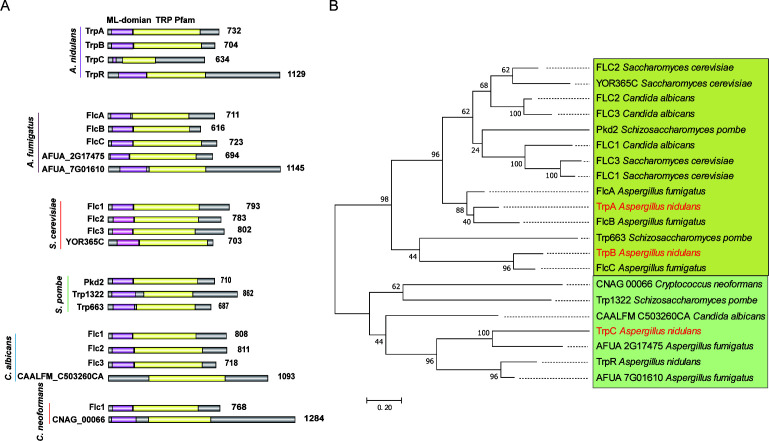
The common shared TRP and other domains in selected putative TRP members and their phylogenetic relationships. (**A**) The illustration shows the functional domain composition of the TRPs with similarly conserved domains in the selected fungal species. The ML domain is shown in pink and the TRP domain is shown in yellow. (**B**) Phylogenetic relationships of the TRPs in the selected species. The yellow or green background represents two different groups. Yellow for subgroup A and green for subgroup B, respectively. The putative TRP proteins in *A. nidulans* are highlighted in red. Phylogenetic analysis was performed using the maximum likelihood method with bootstrap support in MEGA 7 software.

Using the TRP Pfam (PF06011) against the whole genome of *A. nidulans*, we previously found four putative TRP-like proteins. Among these, the biological function of AN9146, referred to as *trpR*, has been verified in a previous study (36). However, the functions of the other three putative TRP members in *A. nidulans* have not been explored yet. According to the SMART interface analysis (http://smart.embl-heidelberg.de/), we found that all putative TRP members shared a common TRP domain and also contained an ML or TRP_N domain (PF14558) (Fig. 1A). Since there were no consistent homolog names referred to in functional known yeasts, we then assigned names to three unknown genes for the following studies: AN1950 (referred to as *trpA*), AN7443 (*trpB*), and AN5367 (*trpC*).

To further explore the homologs of TrpA, TrpB, and TrpC in the most common model and pathogenic fungi, the analysis as aforementioned strategy was performed (Fig. 1A). The selected fungal species exhibit varying numbers of TRP members, such as five TRP members in *A. fumigatus,* four TRPs in *S. cerevisiae,* three TRPs in *S. pombe,* four TRPs in *C. albicans*, and two TRPs in *C. neoformans*. As predicted, all members showed a similar domain pattern with a TRP Pfam (PF06011) and an ML or TRP_N domain, suggesting that these conserved domains of putative TRP-like proteins may play important and conserved functions in the long-term evolution of fungi.

To further explore the evolutionary relationships among these TRP domain-containing proteins, we constructed a phylogenetic tree using amino acid sequences of the TRP domain from these proteins (Fig. 1B). Phylogenetic analysis revealed that these selected TRP domain-containing proteins were divided into two subgroups, namely, subgroup A, comprising TrpA and TrpB, and subgroup B, encompassing TrpC. This suggests that TrpA and TrpB might be more related to each other than to TrpC. Subgroup A (highlighted in yellow) mainly contains members of the FLC family, which is a family of integral membrane proteins and has been named for its crucial role in FLC.

By comparison, TRP domains in TrpA and TrpB share 34%–40% and 23%–25% identity, respectively, with those of the FLC proteins in *S. cerevisiae* and *C. albicans*. Notably, TrpA shows homology to FlcA and FlcB of *A. fumigatus*, exhibiting a high degree of similarity in their TRP domains (72.65% and 57.59%, respectively). This suggests that TrpA might perform both FlcA and FlcB functions, which have been shown to be associated with virulence in *A. fumigatus*. TrpB shares homology with FlcC in *A. fumigatus*, with a 66.05% identity in the TRP domain. On the other hand, TrpC and TrpR in *A. nidulans* belong to subgroup B (highlighted in green). Interestingly, this subgroup B includes the relatively known function of Trp1322 in fission yeast *S. pombe*. This implies that unknown TrpC, despite being relatively unknown, may share similar functions with TrpR and yeast Trp1322.

### Localization of TrpA, TrpB, and TrpC in hyphal cells

**Fig 2:**
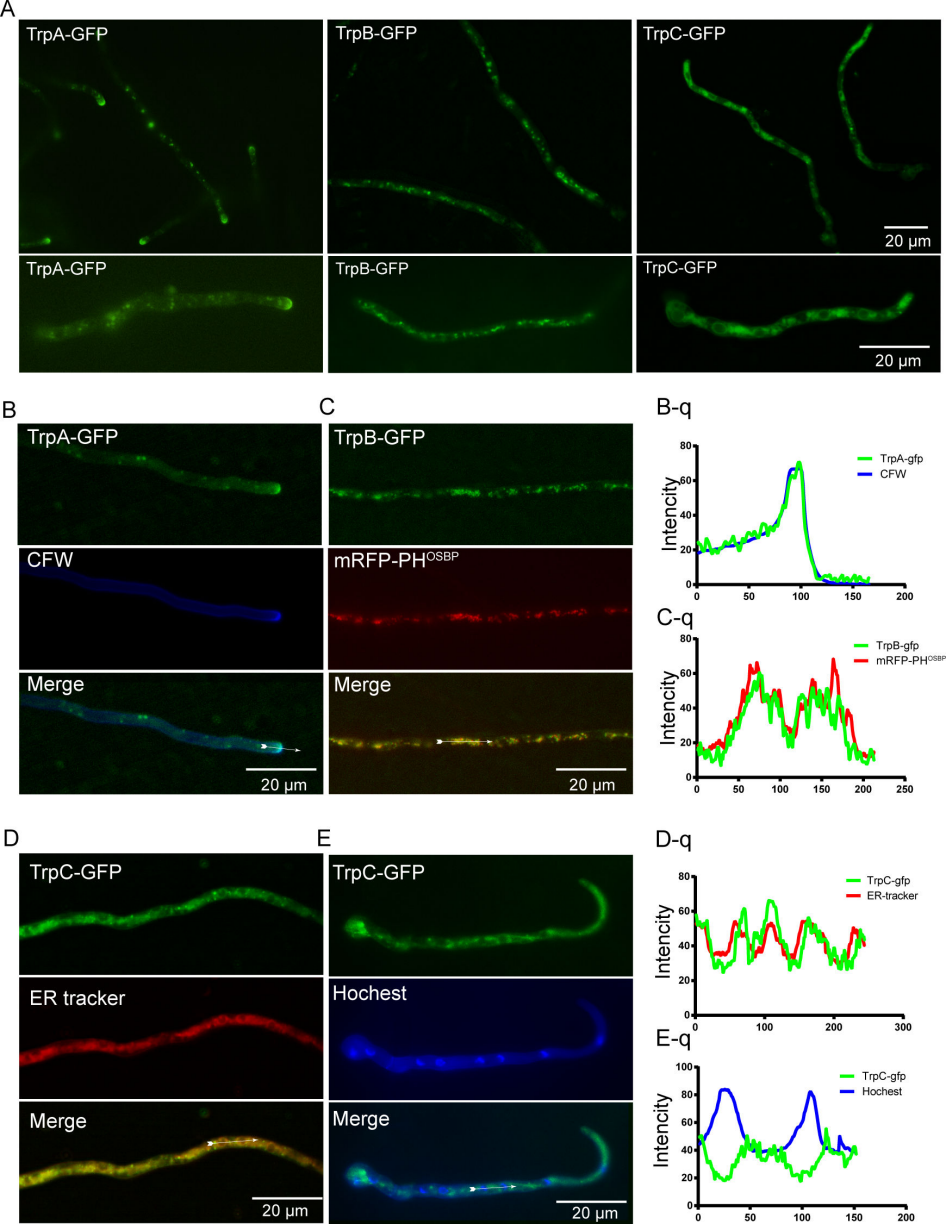
Characterization of the subcellular localization of TrpA, TrpB, and TrpC. (**A**) Epifluorescence distributions of TrpA-GFP, TrpB-GFP, and TrpC-GFP, respectively. (**B**) Epifluorescence localization of TrpA-GFP colocalizes with the chitin aggregation at the hyphal tip. Cells were stained with CFW. (**B–**q) Relative to panel B for the quantitative analysis of the two fluorescence intensities of TrpA-GFP and CFW staining indicated by the arrow. (**C**) Distribution of TrpB-GFP relative to the late Golgi marker PH^OSBP^, demonstrating that TrpB colocalizes with the Golgi marker PH^OSBP^. (**C–**q) Relative to panel C for the quantitative analysis of the fluorescence intensities of TrpB-GFP and mRFP-PH^OSBP^ indicated by the arrow. (**D**) Microscopic observation of TrpC-GFP stained with the ER-Tracker Red, a fluorescent probe for the ER, showed that the distribution of TrpC was consistent with the stained. (**D–**q) Relative to panel D for the quantitative analysis of the fluorescence intensities of TrpC-GFP and ER-Tracker Red staining indicated by the arrow. (**E**) Microscopic observation of TrpC-GFP stained with the blue fluorescent DNA stain Hoechst indicated that TrpC was distributed around the nucleus. (**E–**q) Relative to panel E for the quantitative analysis of the fluorescence intensities of TrpC-GFP and Hoechst staining indicated by the arrow. Scale bar: 20 µm. Hyphae in all panels were analyzed by epifluorescence microscopy after a 10-h incubation in liquid medium MMPDRUU at 37°C.

To further verify the subcellular localizations of these three proteins, we successfully generated the TrpA-GFP, TrpB-GFP, and TrpC-GFP strains by fusing the green fluorescent protein (GFP) to the C-termini of these three TRP proteins. Similar colony growth phenotypes to their parental wildtype suggested that the GFP labeling in these three TRP proteins did not affect the functions of these proteins (Fig. S1A). TrpA-GFP was primarily located at the tip of hyphae, accompanied by a diffuse distribution in the cytoplasm (Fig. 2A). The localization pattern of TrpA closely resembled the distribution pattern attached to the cell wall at the tip of vigorously growing hyphae. To further verify the localization of TrpA, we used calcofluor white (CFW), a chitin-binding reagent, to stain the TrpA-GFP strain (Fig. 2B). Fluorescence observation revealed a pattern of TrpA attached to the chitin aggregation stained by CFW at the hyphal tip. These data suggested that highly tip-localized TrpA likely plays a key role in hyphal polarity growth, potentially related to the accumulation of chitin synthases. The fluorescence localization of TrpB-GFP was observed in punctuated locations within the cytoplasm (Fig. 2A). To further analyze the localization of the TrpB protein, a red fluorescent probe mRFP-PH^OSBP^ (the human oxysterol-binding protein PH domain [37]) was used as a reference for the late/trans-Golgi compartment (Fig. 2C). The results showed that there was a significant colocalization between TrpB-GFP and the Golgi marker mRFP-PH^OSBP^. This observation strongly suggested that TrpB may be localized on the Golgi complex.

In comparison, the TrpC protein displayed a weak distribution pattern similar to that of the ER in the cytosol (Fig. 2A). To further investigate the localization of the TrpC protein, a red fluorescent dye to label the ER was utilized to stain the TrpC-GFP strain (Fig. 2D). The results indicated a partial overlap in content between the regions labeled by the ER-Tracker and those labeled by TrpC-GFP. Furthermore, nuclei were labeled with Hoechst, a nuclear fluorescent dye (Fig. 2E) revealing that the nuclei were mainly located at the center of those labeled with TrpC-GFP. These data suggested that TrpC was likely localized to the ER. Collectively, these data indicated that the aforementioned three putative TRP channels had distinct localization patterns.

### Colony phenotypic characterizations of TrpA, TrpB, and TrpC mutants

**Fig 3:**
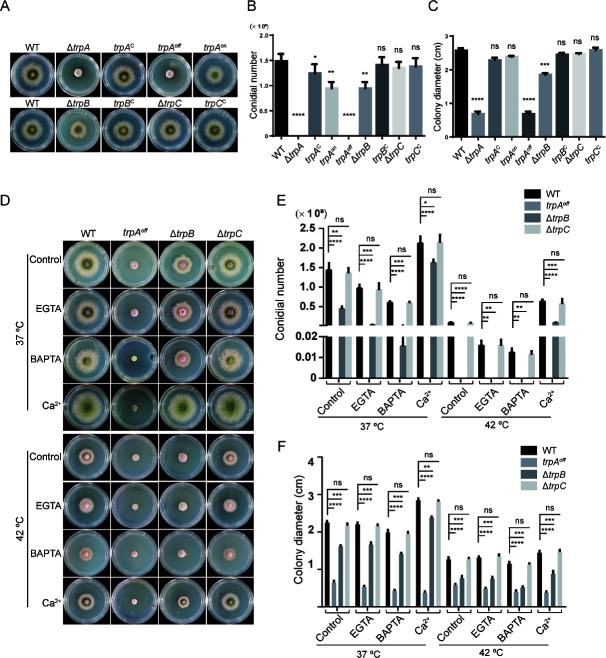
Colony phenotypic characterization of TrpA, TrpB, and TrpC mutants. (**A**) Colony morphology of the mutants and reference wild-type strain on solid MMPDRUU at 37°C for 2 days. (**B and C**) Quantitative conidial numbers and colony diameter for the indicated strains relative to panel A. (**D**) Colony morphology of the mutants and reference wild-type strain on solid MMPDRUU supplemented with 4 mM ethylene glycol tetraacetic acid (EGTA), 85 µM BAPTA-AM, or 50 mM Ca^2+^ at 37°C and 42°C cultured for 2 days, respectively. (**E and F**) Quantitative total conidial numbers and colony diameter for the indicated strains relative to panel D. Values represent mean ± SD from three replicates (ns, not significant; *P < 0.05; **P < 0.001; ***P < 0.001; and ****P < 0.0001).

To better understand the functions of TrpA, TrpB, and TrpC in *A. nidulans*, we constructed three null mutants by replacing relative genes with the *pyrG* selectable marker in the parental strain TN02A7, respectively. The successful generation of these three mutants was confirmed through diagnostic PCR (Fig. S1B). The ∆*trpA* mutant exhibited a severely defective colony phenotype on the solid medium, with a colony diameter of about 30% of the wildtype and nearly complete inhibition of conidiation (Fig. 3A through C). This suggested a crucial role for the TrpA in hyphal growth and conidiation during colony development. The ∆*trpB* mutant displayed a colony diameter around two-thirds smaller and only half the conidial production compared to the wild-type strain on the minimal medium, indicating the significance of TrpB in colony growth and conidiation. However, unlike ∆*trpA* and ∆*trpB*, the ∆*trpC* mutant showed no significant differences in colony diameter and conidial production compared to the wild-type strain on the tested solid medium. In order to confirm that the observed phenotypes were indeed caused by specific gene deletions, we complemented the null mutant strains by reintroducing equivalent genes *trpA*, *trpB*, and *trpC*, respectively. Complementation experiments (*trpA*^c^, *trpB*^c^, and *trpC*^c^), respectively, demonstrated that reintroducing the equivalent genes resulted in phenotypes similar to the parental wildtype. Given the difficulty in harvesting conidia from the *trpA* null mutant for further functional testing, it was challenging to verify whether the expression of *trpA* was associated with the aforementioned pathological phenotypes of the ∆*trpA* mutant. Therefore, we constructed a conditional mutant *alc-trpA*, utilizing the alcohol promoter *alc*, which is repressed by glucose but can be normally expressed when glycerol is the carbon source. Conidia of this strain could be obtained on glycerol medium MMPGR, while functional tests were performed on glucose medium MMPDR. Real-time PCR further confirmed that the expression level of the *trpA* gene, under the control of the *alc* promoter, was only 5% of the wild-type level in medium MMPDR, but it reached the wild-type level in medium MMPGR (Fig. S1B). The results demonstrated the successful construction of the conditioned *alc* promoter-control strain. Phenotypic analysis of the *alc-trpA* mutant in the off-state (“*trpA^off^*”) revealed a comparable phenotype to the complete deletion of *trpA* on medium MMPDR (Fig. 3A), repeatedly demonstrating the phenotype was indeed attributed to the defective *trpA*. In comparison, the strain was able to grow and produce conidia normally in the on-state (“*trpA^on^*”) on medium MMPGR.

Given that TRP channels belong to a superfamily of cation channels and play key roles in environmental perception, we proceeded to investigate whether the aforementioned three TRP mutants exhibited sensitivity to high temperature and calcium ions (Fig. 3D through F). The ∆*trpB* mutant displayed the most obvious changes among three different calcium supplementation treatments for tested strains based on the MMPDRUU as a control and ethylene glycol tetraacetic acid (EGTA) and 1,2-bis(o-aminophenoxy)ethane-N,N,N′,N′-tetraacetic acid tetrakis(acetoxymethylester) (BAPTA-AM) as calcium chelators. When cultured at 37°C, ∆*trpB* showed defective colony phenotypes in a calcium-limited dependent way. In the presence of EGTA or BAPTA-AM, virtually no conidial production was detected, but the addition of calcium significantly rescued these deficiencies. Notably, when ∆*trpB* was cultivated on the minimal medium (control) or supplemented with EGTA or BAPTA-AM at 42°C, it displayed minute and fluffy colonies, exhibiting considerably more severe defective phenotypes compared to the corresponding media at 37°C. These data suggested that TrpB plays an important role in colony development under calcium-limited and high-temperature-stress conditions.

However, under all tested conditions, the *trpA^off^* mutant consistently exhibited diminutive and fluffy colonies, indicating the indispensable requirement of TrpA for these conditions, with no tested condition being able to circumvent the need for TrpA. In comparison, no detectable differences were observed in the ∆*trpC* mutant compared to the parental wild-type strain under the above conditions.

### TrpA and TrpB but not TrpC play important roles in regulating cellular calcium homeostasis under liquid cultural conditions

**Fig 4:**
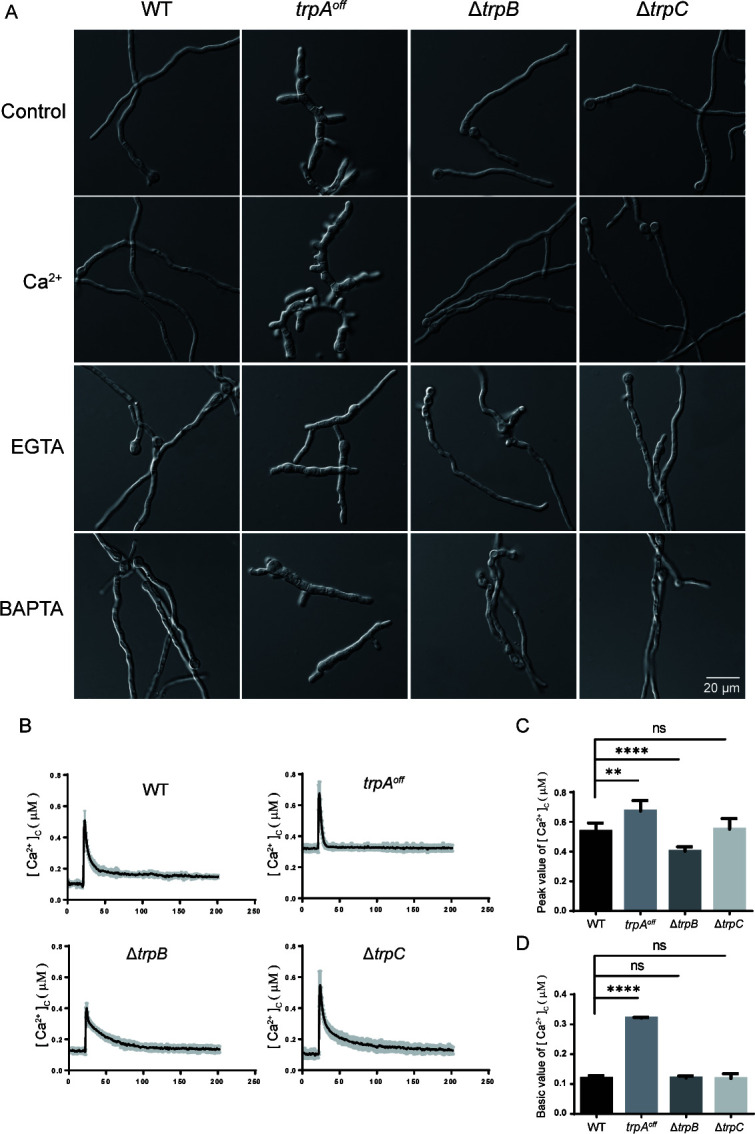
TrpA and TrpB, but not TrpC, play crucial roles in regulating cellular Ca^2+^ homeostasis. (**A**) Hyphal morphology of the indicated strains under liquid MMPDRUU medium, treated with CaCl_2_ (40 mM), EGTA (4 mM), and BAPTA-AM (85 µM) at 42°C for 12 h. Scale bar: 20 µm. (**B**) Real-time monitoring of the cytosolic calcium concentration ([Ca^2+^]_c_) in the indicated strains following stimulation with 20 mM CaCl_2_. (**C **and **D**) Quantitative of the peak and baseline of transient [Ca^2+^]_c_ in the indicated strains as shown in panel C. Values represent mean ± SD from three replicates (ns, not significant; **P < 0.01 and ****P < 0.0001).

To further verify the effect of these three TRP mutants on the regulation of cellular calcium homeostasis, we separately analyzed the hyphal morphology of the corresponding mutants under liquid conditions (Fig. 4A). At 42°C, the *trpA^off^* mutant displayed diminished hyphal lengths compared to the wild-type strain. Surprisingly, an increased branching phenomenon in *trpA^off^* was observed with a more severe tendency in the calcium-added environment (40 mM) than in the control MMPDRUU. In contrast, under the calcium-limited condition achieved by adding EGTA or BAPTA-AM, the branching of the *trpA^off^* strain was significantly reduced and the abnormal hyphal polarity was significantly rescued. These results suggested that reduced expression of TrpA is associated with cytosolic Ca^2+^ imbalance and may lead to Ca^2+^ toxicity in the cell, and then extracellular calcium limitation was able to rescue defective polarity growth of germlings in the *trpA^off^* mutant. In comparison, abnormal germling morphology was observed in ∆*trpB* under the calcium-limited condition compared to the wild-type strain, while adding calcium was able to remarkably rescue the aforementioned abnormalities. However, ∆*trpC* did not exhibit any abnormal characterization under either calcium-supplemented or calcium-limited liquid culture conditions.

To further investigate the impact of calcium concentrations on these mutants, we examined the characterizations of submerged fermentation for TRP mutants and wild type (Fig. S2). At 37°C, mycelium pellets of *trpA^off^* were much smaller and more granular than those of the wild-type strain across all tested media. Particularly, when cultured in calcium-rich media, *trpA^off^* showed reduced biomass production and exhibited a yellowish coloration, suggesting that turning off the expression of TrpA not only hinders mycelial development but also potentially leads to abnormal secondary metabolism. In contrast, ∆*trpB* exhibited a further decrease in biomass and a brown-yellowish color under low calcium conditions, indicating that the absence of TrpB disrupts metabolic processes and hinders hyphal growth. However, no detectable differences were observed between ∆*trpC* and its parental wild-type strain under the tested cultural conditions.

To gain a better understanding of whether these three TRP channels are involved in cellular responses to calcium stimulation, we monitored the concentration of cytosolic Ca^2+^ ([Ca^2+^]_c_) transient induced by extracellular calcium in living cells, using aequorin as a calcium-sensing reporter (Fig. 4B through D). The resting [Ca^2+^]_c_ of the wild-type strain was approximately 0.11 µM. Upon stimulation with Ca^2+^ (20 mM), the [Ca^2+^]_c_ transiently increased from the resting state to a peak concentration of approximately 0.6 µM. In comparison, the resting [Ca^2+^]_c_ of *trpA^off^* was significantly higher than that of the wildtype, reaching 0.32 µM, twice the value of the wildtype. These data suggested that TrpA plays a crucial role in maintaining [Ca^2+^]_c_, implying that the absence of TrpA may influence the balance of intracellular Ca^2+^. In comparison, there was no significant difference between the ∆*trpB* mutant and the wild-type strain in the resting state. However, when stimulated with calcium, the ∆*trpB* mutant showed a reduction in the transient peak [Ca^2+^]_c_ by approximately 60% compared to that of the wild-type strain, which was about 0.4 µM, suggesting that the loss of TrpB results in reduced responses to the calcium stimulus. For the ∆*trpC* mutant, there were also no detectable changes compared to the wild-type strain, either at rest or when stimulated with calcium. Since TrpC is localized in the ER, we hypothesized that it may have a function in response to ER stressors. To verify this, we utilized tunicamycin (TM), a specific inducer of ER stress, to stimulate both the ∆*trpC* mutant and wild-type strains (Fig. S3). The transient peak [Ca^2+^]_c_ *of ∆trpC* was significantly lower than that of the wildtype, reaching only 60% of its concentration when treated with 5.65 nM TM combined with 1 mM EGTA, demonstrating that the lack of TrpC caused a decreased transient peak [Ca^2+^]_c_ response to ER stress when blocking extracellular calcium entry with EGTA. These data suggested that TrpC may be involved in functions related to ER stress responses.

### TrpA, but not TrpB and TrpC, is involved in chitin biosynthesis of cell walls

**Fig 5:**
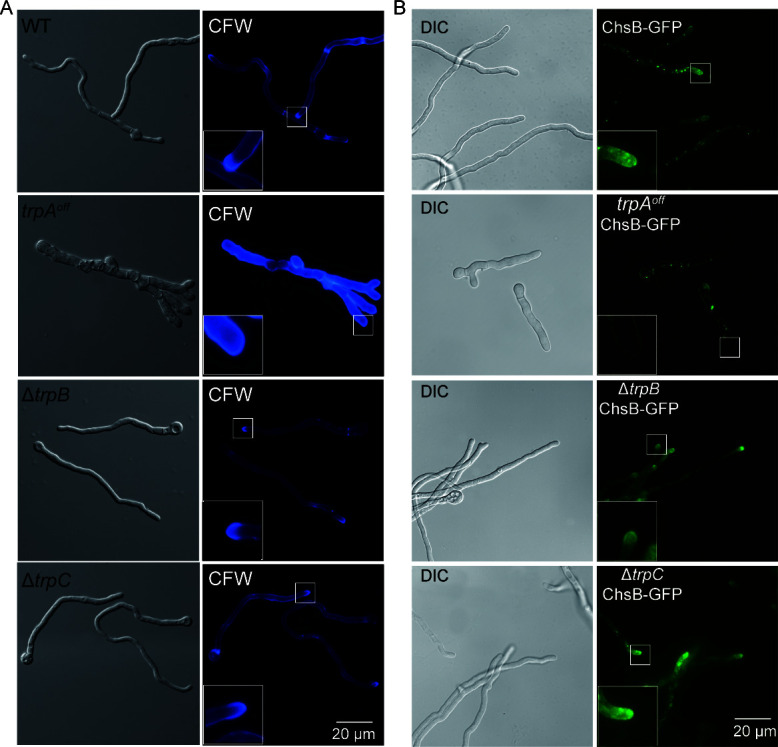
TrpA, but not TrpB and TrpC, is involved in the chitin biosynthesis of cell walls. (**A**) The distribution of the cell wall in the indicated strains was visualized through fluorescence microscopy using CFW staining. The hyphae were cultured at 37°C for 10 h and stained by 1 µM CFW for 5 min. The corresponding hyphae are shown on the left in the form of differential interference contrast (DIC) microscopic images. Scale bar: 20 µm. (**B**) Epifluorescence observations compare the localizations of the chitin synthetase ChsB in the wild-type strain and three TRP mutants. The hyphae were cultured at 37°C for 10 h. DIC microscope image of relative labeled strains on the left. Scale bar: 20 µm.

Hyphal morphogenesis (polar growth) in *A. nidulans* is mainly dependent on the cell wall components composed of glucan and chitin. To explore whether the loss of these three TRP channels impacts the cell wall, we examined the distribution of cell wall chitin by staining it with the chitin-binding agent CFW (Fig. 5A). The wild-type strain exhibited a uniform distribution of chitin throughout the cell wall, with a crescent-shaped aggregation of chitin at the hyphal tip. In contrast, the *trpA^off^* mutant showed significantly intensified CFW staining and loss of the characteristic chitin aggregation at the hyphal tip compared to the wild type. These findings suggested that TrpA is required for the normal distribution of cell wall materials. However, no abnormal chitin distribution was observed in the ∆*trpB* and ∆*trpC* mutants.

To further investigate the effect of TrpA on the hyphal cell wall, we tagged the chitin synthetase gene *chsB*, which is primarily responsible for chitin synthesis, with GFP (Fig. 5B). In the wild-type strain, ChsB mainly accumulated at hyphal tips, as anticipated. However, the *trpA^off^* mutant exhibited a markedly distinct localization of ChsB, losing its apical localization and distribution throughout the cytoplasmic membrane. This suggested that the suppression of TrpA expression leads to an abnormal distribution of the chitin synthetase ChsB. No significant abnormality in ChsB localization was found in ∆*trpB* and ∆*trpC* mutants. Considering the high temperature-sensitive phenotype of the ∆*trpB* mutant, the distribution of ChsB in the ∆*trpB* mutant was examined at 42°C. No significant abnormalities were found compared to the wild-type strain (Fig. S4). Furthermore, when examining the sensitivity of these three mutants to cell wall disruption reagents, the *trpA^off^* mutant showed increased tolerance to such reagents (Fig. S5), while the sensitivity of the *∆trpB* and ∆*trpC* mutants remained similar to that of the wild type. These data collectively emphasized that TrpA, but not TrpB and TrpC, may be involved in the biosynthesis of cell walls.

### Colony phenotypic comparisons in double mutants of TrpA with TrpB or TrpC and with well-known calcium channels

**Fig 6:**
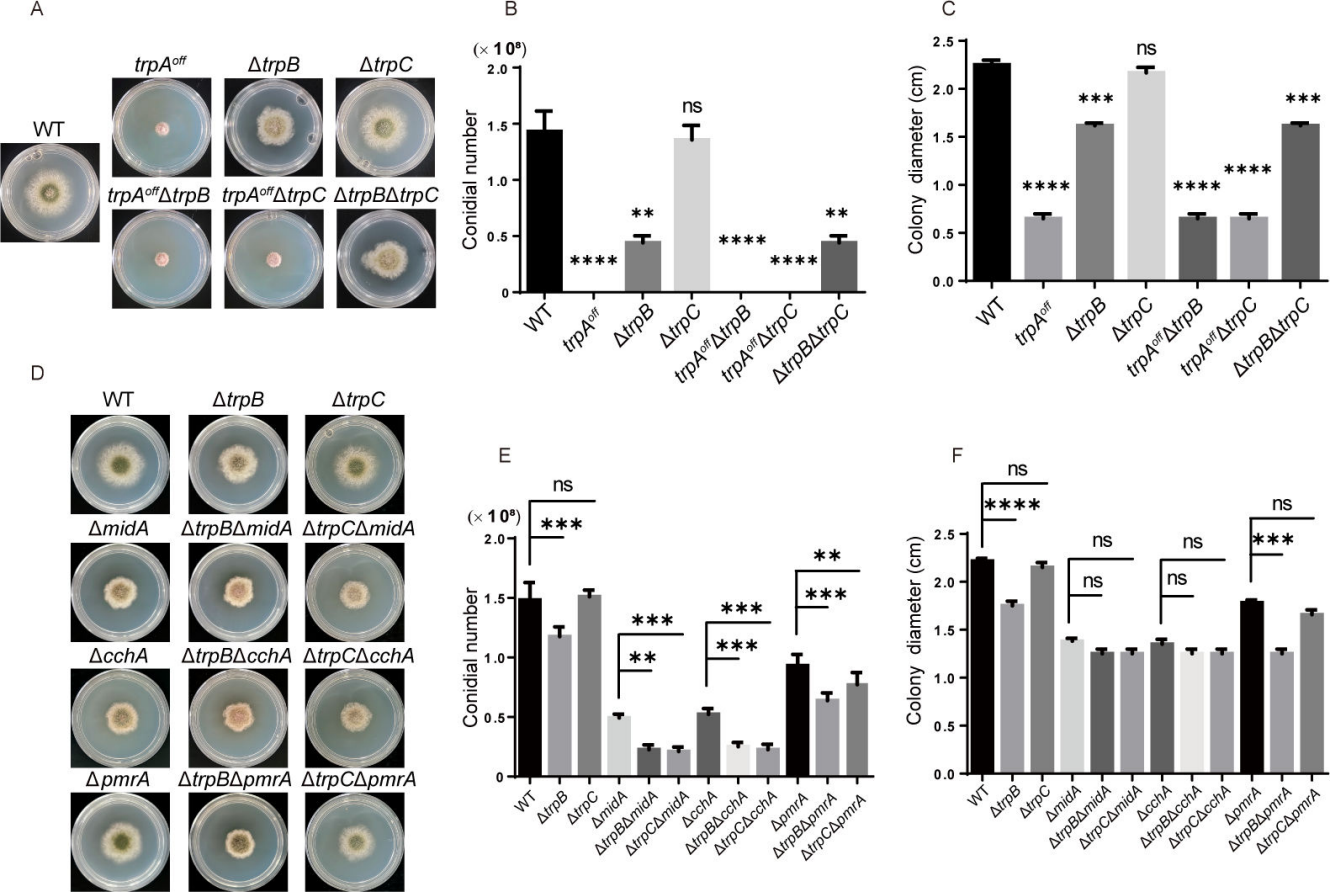
Comparison of colony phenotypes in a double mutant of TrpA with TrpB or TrpC and well-known calcium channels. (**A** and **D**) Comparison of colony morphology for the indicated strains grown on solid MMPDRUU at 37°C for 2 days. (**B**, **C**, **E**, and **F**) Quantification of conidial numbers and colony diameters for the strains relative to panels **A** and **D**, respectively. Error bars represent the standard deviations from three replicates (ns, not significant; *P < 0.05; **P < 0.001; ***P < 0.001; and ****P < 0.0001).

Given the previous data indicating that TRP channels are involved in the regulation of cellular calcium, we sought to investigate whether there is a synergistic relationship between TrpA, TrpB, and TrpC, as well as between these proteins and other calcium channel proteins. To investigate the relationship between these three proteins, we constructed three double mutants: *alc-trpA*∆*trpB*, *alc-trpA*∆*trpC*, and ∆*trpB*∆*trpC* via a genetic crossing strategy. The colony morphologies of *trpA^off^*∆*trpB* and *trpA^off^*∆*trpC* double mutants were identical to that of the *trpA^off^* mutant on minimal medium MMPDRUU (Fig. 6A through C), suggesting that TrpA is important and may function separately and independently from TrpB and TrpC, without any overlap or suppression in the double mutants. In addition, ∆*trpB*∆*trpC* also exhibited a colony morphology similar to ∆*trpB*. These results suggested that these three TRP channels may not compensate for or overlap with each other.

To investigate the correlation between these TRP channels and the well-known calcium channels in *A. nidulans*, we selected the high-affinity calcium system—CchA/MidA and the P-type Golgi Ca^2+^ ATPase PmrA deletion mutants by crossing with Trp mutants, relatively. A series of corresponding double deletion mutants were successfully generated, and their colony phenotypes were analyzed (Fig. 6D through F). Consistent with previous reports, the ∆*midA*, ∆*cchA*, and ∆*pmrA* mutants displayed smaller colony sizes and a decreased number of conidia compared to the wild-type strain on MMPDRUU. In comparison, the ∆*trpB*∆*midA*, ∆*trpB*∆*cchA*, and ∆*trpB*∆*pmrA* double mutants all exhibited aggravated defects than their parental single deletion mutants. This may indicate that there is a synergistic effect of TrpB with calcium channels—MidA, CchA, and PmrA. Surprisingly, although ∆*trpC* single deletion had no detectable phenotype, the ∆*trpC*∆*midA*, ∆*trpC*∆*cchA*, and ∆*trpC*∆*pmrA* double mutants also exhibited exacerbated defects compared to their parental channel deletion mutants, suggesting a compensatory effect for TrpC in the absence of MidA, CchA, and PmrA as well. Notably, the phenotypes of all the aforementioned mutants can be effectively restored by the addition of calcium (Fig. S6), implying that the addition of abundant calcium can bypass the requirement for all tested mutants.

### Comparative transcriptomic analysis in TrpA, TrpB, and TrpC mutants compared to the parental wild-type strain

**Fig 7:**
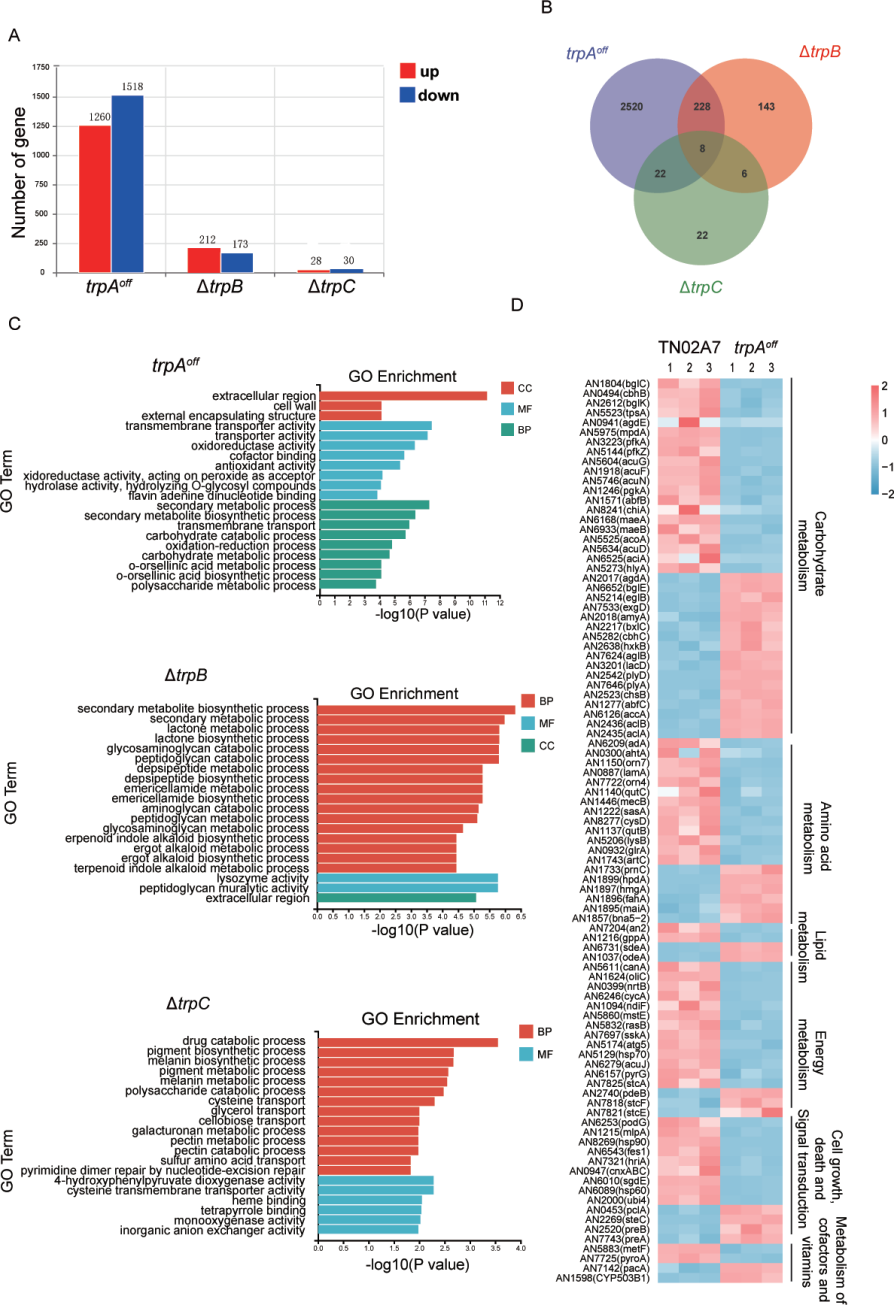
RNA-seq analysis reveals potential functions of TrpA, TrpB, and TrpC. (**A**) Statistics of significantly differentially expressed genes (DEGs) in *trpA^off^*, ∆*trpB*, and ∆*trpC* mutants. Expression difference multiples |log_2_fold change| > 1 and false discovery rate *P*-value < 0.05. (**B**) Venn diagrams of the DEGs in *trpA*^off^, ∆*trpB*, and ∆*trpC* mutants showing overlaps and distinct sets. (**C**) The most significantly enriched gene ontology (GO) molecular functions (38), cellular components (CC), and biological processes (BP) are shown for the DEGs of *trpA^off^*, ∆*trpB*, and ∆*trpC* mutants. (**D**) Heat map comparison of the RNA-seq data in the *trpA^off^* mutant compared to the wild-type strain. Colors represent changes in fpkm values of the RNA levels detected in the indicated strains.

To further understand the function of TrpA, TrpB, and TrpC, we performed transcriptome sequencing (RNA-seq) on three biological replicates and compared the transcriptomes of the *trpA^off^*, ∆*trpB*, and ∆*trpC* mutants with the wild-type strain, respectively. The expression difference of multiples |log_2_fold change| > 1 with a false discovery rate *P*-value < 0.05 was used as the condition for screening differentially expressed genes (DEGs) (Fig. 7A). The total number of upregulated genes in the *trpA^off^*, ∆*trpB*, and *∆trpC* mutants was 1,260, 212, and 28, respectively. Additionally, the number of downregulated genes was 1,518, 173, and 30, respectively, when compared to the parental wild-type strain. These findings suggest that the reduced expression of TrpA (about 10% of wild type) in the *trpA^off^* strain resulted in a large number of gene expression changes (about one-third of all genes), implying that TrpA may serve as a global regulator in *A. nidulans*. In comparison, the absence of TrpB caused significant changes in the expression of 385 genes, while the absence of TrpC only induced changes in the expression of 58 genes. By counting the number of common and unique DEGs among these three mutant strains and comparing the overlap of common DEGs between them (Fig. 7B), we found that only eight DEGs were shared among the *trpA^off^*, ∆*trpB*, and ∆*trpC* mutants, suggesting that the genes regulated by these three TRP channels were relatively independent of each other. However, there were a considerable number of DEGs shared between the *trpA^off^* and ∆*trpB* mutants, which accounted for 61% of the total number in the ∆*trpB* mutant. Additionally, 52% of the total DEGs of the ∆*trpC* mutant were shared with those in the *trpA^off^* mutant, indicating that TrpA may have partially similar functions to TrpB and TrpC. However, TrpA appears to play a more predominant role.

Gene ontology (GO) enrichment analysis was performed according to the DEGs of these three mutants, further suggesting their potential involvement in distinct pathways (Fig. 7C). Notably, the downregulation of TrpA affected a variety of metabolic processes, including the metabolism of carbohydrates, amino acid, lipid, and energy, among others. It suggested that TrpA has a wide range of effects on physiological processes. Furthermore, the heat map displayed significant changes in gene expression of *trpA^off^* compared to that of the wildtype according to Kyoto Encyclopedia of Genes and Genomes database enrichment analysis (Fig. 7D). Regarding the function of TrpB (Fig. 7C), the GO analysis indicated that it may be involved in the biosynthesis and secondary metabolism of various cellular secondary metabolites, including lactone, glycosaminoglycan, peptidoglycan, and depsipeptide. In addition, molecular function predictions suggested that TrpB may possess lysozyme and peptidoglycan hydrolysis activities. While no evident effect on growth and conidiation processes was observed in the ∆*trpC* mutant, transcriptome analysis revealed the potential involvement of TrpC in drug catabolic processes (Fig. 7C). In addition, TrpC may also be involved in the biosynthesis and metabolism processes of pigments and melanin.

Given that the aforementioned data indicate the involvement of TrpA, TrpB, and TrpC in the regulation of cellular calcium equilibrates, we then screened annotated genes associated with calcium ion transport or calcium metabolism among DEGs in the three TRP mutants. Finally, the expression levels of seven calcium regulatory proteins were identified (Table 1). The expression levels of six calcium regulators in the *trpA^off^* mutant were increased to varying degrees compared to the wild-type strain, including the high-affinity calcium channel proteins MidA and CchA, as well as the ATP-type calcium transporters PmcA and PmcB. However, the expression level of the potential calcium transporter, AN7286, identified in the *∆trpB* mutant, was decreased compared to the wild-type strain but not found in the Δ*trpC* mutant.

**TABLE 1:** List of differentially expressed genes associated with calcium transport in *trpA^off^* and ∆*trpB*

Strain	Gene ID	Product description	log_2_fold change (mutants/WT)
*trpA^off^*	AN1168 (*cchA*)	Putative voltage-gated calcium channel	1.547464591
*trpA^off^*	AN1189 (*pmcA*)	Putative calcium-transporting vacuolar ATPase involved in calcium homeostasis	1.164142195
*trpA^off^*	AN4920 (*pmcB*)	Putative calcium-transporting mitochondrial ATPase involved in calcium homeostasis	1.098711205
*trpA^off^*	AN5821 (-)	Putative vacuolar H^+^/Ca^2+^ exchanger	2.145293257
*trpA^off^*	AN7571 (-)	Ortholog(s) has stretch-activated, cation-selective, calcium channel activity	1.729260135
*trpA^off^*	AN8842 (*midA*)	Putative stretch-activated calcium channel	1.398315026
*∆trpB*	AN7286 (-)	Ortholog(s) has a role in calcium ion import and plasma membrane localization	1.11992

## DISCUSSION

TRP proteins constitute a superfamily that encodes transmembrane ion channels with diverse permeation and gating properties (3, 6, 7). In mammals, TRP channels are primarily recognized as sensors for environmental stimuli that trigger somatosensory responses (39, 40). Moreover, more and more studies have demonstrated that TRP channels play significant roles in many physiological and pathological processes (2–4). As a result, the physiological roles of the TRP family have garnered considerable attention (6). In recent years, homologs of the TRP family have also been found in yeast and other fungal species (39, 40). The first TRP protein confirmed in *S. cerevisiae* is Yvc1p, which is located in the vacuole, and it regulates cytosolic calcium signaling by releasing Ca^2+^ from the vacuole under hyperosmotic stress (14, 16, 41, 42). The activation mechanism of Yvc1p channels has been found to be related to glutathione depletion (42). Moreover, there have also been TRP-like ion channels identified in fungal cells that contain the TRP domain (PF06011). Herein, we identified three potential TRP-like proteins by bioinformatics analysis in the filamentous fungus *A. nidulans*: AN1950 (*trpA or flcA*), AN7443 (*trpB*), and AN5367 (*trpC*). Through gene deletion, GFP tagging, and monitoring cytosolic Ca^2+^ concentration ([Ca^2+^]_c_), we characterized these proteins and verified their roles in hyphal growth, conidiation, and multiple stress responses. We demonstrated that these proteins are involved in the regulation of Ca²^+^ homeostasis. Additionally, we further elucidated the functions of TrpA, TrpB, and TrpC by performing transcriptome sequencing.

TrpA, the most pivotal one among these three proteins, plays crucial roles not only in *A. nidulans* but also in its homologs in other species (22, 29). Our data suggested that the TrpA protein is localized at the hyphal tip (Fig. 2B), akin to the homologous Pkd2 in *S. pombe* and FlcA in *A. fumigatus*, as reported in other previous studies (20, 29). We found that the deletion of TrpA resulted in severe colony growth defects, almost abolishing conidiation, demonstrating TrpA is required for colony development, but it is not essential for survival in *A. nidulans*. By constructing the conditional strain *trpA^off^*, we discovered that reduced TrpA expression led to comparably defected phenotype akin to the *trpA* null deletion strain, suggesting that normal expression of TrpA is required for hyphal growth and conidiation (Fig. 3A). In addition, microscopic observation showed that the *trpA^off^* mutant exhibited abnormally increased hyphal branching with lost hyphal polarity. Notably, data found in this study provided repeated evidence that the increased branching of hyphae induced by TrpA downregulation is closely related to the recovery of cytosolic Ca²^+^ concentration to the normal status since high calcium conditions are able to exacerbate the abnormality (Fig. 4A). Conversely, the defect in hyphal branching was significantly alleviated in the presence of the calcium chelator EGTA and BAPTA-AM. These findings suggested that TrpA plays a crucial role in regulating Ca²^+^ balance. Moreover, by monitoring the [Ca^2+^]_c_, we observed that a decrease in *trpA* expression led to a significant increase in cytosolic Ca²^+^ concentration compared to the wild-type strain (Fig. 4B). Thus, it suggested that deficiency of TrpA severely disrupts intracellular Ca²^+^ homeostasis, ultimately resulting in the toxic effect of high Ca²^+^ concentration on the *trpA^off^* mutant. However, regardless of whether under high or low calcium cultural conditions, deficiency of TrpA always displayed significantly reduced colony size, fewer conidia numbers, and decreased biomass compared to the wildtype (Fig. 3D; Fig. S2B), suggesting that apart from regulating cellular Ca^2+^ homeostasis, TrpA may have multiple functions required for hyphal growth and reproduction. Accordingly, a comparative transcriptomic analysis between the *trpA^off^* mutant and the wildtype verified that TrpA is a global regulator. Reduced expression of TrpA resulted in a large number of changes in gene expression in *A. nidulans* (Fig. 7A). Previous studies have found the TrpA putative homologs FlcA and FlcB in *A. fumigatus*, which play crucial roles as flavin carriers. Thus, TrpA may function as a flavin carrier in a calcium-regulating independent way. Besides, the TRP-like ion channel Pkd2 (TrpA homolog in *S. pombe*) has been identified as a Ca^2+^-permeable channel and is responsive in regulating cytoplasmic Ca^2+^ homeostasis (19). In addition, our data also demonstrated that TrpA plays an important role in regulating the proper distribution of chitin in the cell wall and the correct localization of chitin synthetase ChsB (Fig. 5). These data suggested that TrpA may contribute to the disruption of cell wall synthesis by affecting the polar transport of substances.

TrpB was verified to be colocalized with the Golgi marker mRFP-PH^OSBP^ (Fig. 2C) and plays crucial roles in colony growth and conidiation, especially under calcium-limited and high-temperature cultural conditions (Fig. 3D). Our data showed that the absence of TrpB leads to restricted colony growth and reduced conidiation and makes the strain highly sensitive to low calcium conditions. Moreover, the defects of the Δ*trpB* mutant are further exacerbated at 42°C. These data suggested that TrpB may function as a Ca^2+^ channel or a temperature sensor, and its role is particularly crucial under high temperatures. However, these defects in the Δ*trpB* mutant can be significantly improved by the addition of calcium, indicating that the loss of TrpB may result in a deficiency of Ca^2+^. However, unlike in other species, FlcC, the homolog of TrpB in *A. fumigatus*, did not play a significant role in colony growth and conidiation (28, 29). This suggested that putative TRP homologs may have their unique species-specific functions, even within the same genus *Aspergillus*. In addition, analysis of the [Ca^2+^]_c_ showed that the transient peak of cytosolic Ca²^+^ concentration induced by high calcium stimulation in the Δ*trpB* mutant was much lower than that in the wild-type strain (Fig. 4B). This further indicated the involvement of TrpB in the cellular response to the regulation of Ca²^+^ homeostasis in hyphal cells. Through transcriptome sequencing, we discovered that TrpB may have functions in biosynthesis and secondary metabolism of various cellular secondary metabolites (Fig. 7C).

Deletion of TrpC did not lead to any abnormal phenotypes under different selected test conditions, such as low calcium condition, high temperature, or exposure to cell wall stress agents (Fig. 3D; Fig. S5). This suggested that TrpC may function as a redundant gene, operating only under certain specific conditions. Fluorescence observation verified that TrpC might be localized in the ER (Fig. 2D and E). Additionally, when stimulated by TM, an ER stress agent, under low calcium conditions, the transient peak of cytosolic Ca²^+^ concentration in the Δ*trpC* mutant was much lower compared to that of the wildtype (Fig. S3). Therefore, we deduced that TrpC also has the capacity to regulate Ca²^+^, but its function can only be reflected under certain specific conditions. Nevertheless, through an investigation of the relationship between these TRP channels and the well-known calcium channels, MidA/CchA and PmrA (Fig. 6), we discovered that the absence of the major high-affinity calcium channels worsened colony defects in both TrpB and TrpC. These data suggested that TrpB, TrpC, and the high-affinity Ca²^+^ channel system have additive compensatory effects.

## MATERIALS AND METHODS

### Strains and growth conditions

The *A. nidulans* strains used in this study are summarized and listed in Table S1. Experiments were performed in minimal medium MMPDR or MMPDRUU (36, 43). The formulations of the media and reagents used in this study are listed and shown in Table S2. Conidia were harvested on the solid medium using sterile H_2_O and stored for long-term preservation in 50% glycerol at −80°C. The conidia of the *trpA^off^* mutant were obtained by incubating them on the MMPGR medium (where glucose in the MMPDR medium was replaced with 2% glycerol) at 37°C.

### Genetic mutant strains’ construction

In this study, gene deletion, complementation, conditional promotor, and GFP tagging were accomplished by the principle of homologous recombination (44), using a selectable marker and the 5′ and 3′ flanking regions of the target locus. Subsequently, these three fragments were combined via fusion PCR and the fusion product was transformed into the recipient strain containing a deletion of the *KU80* gene for efficient site-specific integration. The primers used in this study are listed in Table S3. Fungal transformation was performed as previously described (36).

Taking the construction of Δ*trpA* as an example, we used *pyrG* as a selectable nutritional marker and amplified the 5′ and 3′ flanking regions of the *trpA* open reading frame from *A. nidulans* TN02A7 genomic DNA with primer pairs *trpA* P1/P3 and *trpA* P4/P6, respectively. Finally, the amalgamation of these three fragments was achieved through fusion PCR, using the primer pair *trpA* P2/P5. The resulting PCR product was then transformed into the recipient wild-type strain.

To construct strains expressing mRFP-PH^OSBP^ and aequorin, we transformed the plasmid containing codon-optimized target genes and selective markers into the indicated strains (37, 45). Subsequently, we screened the transformers using diagnostic PCR assays or microscopic testing and selected strains that expressed the desired traits for further experimental studies.

The double mutant strains were generated by crossing, which was described previously (43). Briefly, we inoculated two parent strains on a plate for 2 days at 37°C. Then, the mixed grown hyphae were transferred to a screening medium supplemented with 150 µM riboflavin and incubated for 15 days at 30°C until the cleistothecia was mature. A grain of cleistothecium was picked up, and the surrounding hyphae were carefully cleaned, then put into a 1.5 mL centrifuge tube, and the tube was pricked; following this, 1 mL of sterilized H_2_O was added and thoroughly mixed. Finally, a 10 µL suspension of ascospores was removed and coated with a screening medium for culture.

### RNA extraction and quantitative RT-PCR assays

For RNA extraction, 1 × 10^8^ fresh conidia were inoculated into 100 mL of the liquid medium MMPDRUU or MMPGRUU and incubated for 24 h at 37°C in shaking flasks. Subsequently, the mycelia were harvested and snap-frozen in liquid nitrogen. RNA extraction was performed using an RNAzol RT column kit (Sangon Biotech, B511631-0100). Reverse transcription-PCR and qRT-PCR analyses were conducted using the HiScript II Reverse Transcriptase (Vazyme, R201-01) and SYBR Premix Ex Taq (TaKaRa, DRR041A), respectively. Specific operations followed the instructions outlined in the protocol manual, and the transcription levels were calculated using the comparative threshold cycle (Δ^CT^) method (44).

### Phylogenetic analysis

All amino acid sequences utilized in this study were sourced from the fungal databases FungiDB (http://fungidb.org/fungidb) and NCBI (https://www.ncbi.nlm.nih.gov). The HMM profile for the TRP domain (PF06011) was downloaded from the PFAM protein family database (http://pfam.xfam.org) and used to identify TRP genes from the *A. nidulans* genome with HMMER 3.0 (http://hmmer.janelia.org/) (46). The phylogenetic tree was constructed using the maximum likelihood method with bootstrap support in MEGA 7 software.

### Plate assays

To analyze the colony morphology of different strains, a certain amount of conidia was inoculated on the solid medium. The operation process was as follows: fresh conidia were diluted to 1 × 10^6^ conidia·mL^−1^ with sterile water and then 2 µL of conidia was spotted onto relevant media and incubated at the indicated temperature for 2 days. At least three replicates were performed for each assay. In order to observe the response of strains to different pressures, corresponding pressure reagents were added to the medium, including indicated doses of CaCl_2_ and the calcium ion chelator EGTA (Merck Research Laboratories, Rahway, NJ, USA, CAS No.: 67-42-5) or BAPTA-AM (Sigma-Aldrich, St. Louis, MO, USA, CAS No.: 126150-97-8), as well as the cell wall stress reagents, Congo red (Sigma-Aldrich, St. Louis, MO, USA, CAS No.: 573-58-0), CFW (Sigma-Aldrich, St. Louis, MO, USA, CAS No.: 4193-55-9), and caspofungin (Merck Research Laboratories, Rahway, NJ, USA, CAS No.: HY-17006).

### Microscopic observation

For the observation of hyphal growth, approximately 1 × 10^4^ conidia of the relevant strains were cultivated on slides overlaid with 0.5 mL of the MMPDRUU medium and incubated at the indicated temperature for approximately 10 h. We removed the medium and washed the hypha three times with the phosphate-buffered saline solution. Following, the hyphae were fixed with 4% paraformaldehyde (Beyotime, Shanghai, China, CAS No.: P0099) for about 20 min at room temperature in the dark. The paraformaldehyde was washed off, and the sample was visualized under a fluorescence microscope. CFW and ER-Tracker staining (Beyotime, Shanghai, China, CAS No.: C1041S) were performed according to the protocol manual. Images were captured with a Zeiss Axio Imager A1 microscope (Carl Zeiss, Jena, Germany). The fluorescence intensity of the images was analyzed using ImageJ.

### Cytoplasmic [Ca^2+^]_c_ monitoring

Cytoplasmic Ca^2+^ concentration ([Ca^2+^]_c_) monitoring was performed as previously described (36, 47, 48). The pAEQ vector containing the codon-optimized aequorin gene was transformed into the indicated strains using fungal transformation. The fresh conidia of the strains that successfully expressed the aequorin were inoculated into the liquid MMPDRUU medium, which was adjusted to a concentration of 1 × 10^7^ conidia·mL^−1^. The conidia were then distributed into a 96-well microdroplet plate, with each well containing 100 µL of the conidia solution. After a static culture at 37°C for 20 h, the mycelium was washed twice with PGM solution. Subsequently, it was incubated with 100 µL of a 25 µM coelenterazine solution (Sigma-Aldrich, St. Louis, MO, USA, CAS No.: 55779-48-1) for 4 h in the dark at 4°C, followed by a 1 h revival at room temperature. The LB 96P Microlumat luminometer (Berthold Technologies, Germany) was used for the detection. Finally, the relative light unit values were converted into the [Ca^2+^]_c_ (45).

### RNA-seq analysis

For sample preparation, 1 × 10^8^ fresh conidia from the respective strains were inoculated in 100 mL of liquid MMPDRUU at 37°C for 20 h. Subsequently, the hyphae were collected and rapidly frozen in liquid nitrogen. The work of transcriptome sequencing was performed by Personalbio Company, Nanjing. All transcriptome profiling was carried out using mapping tools available on the genescloud platform (https://www.genescloud.cn).

## SUPPLEMENTAL MATERIAL

Figure S1 msphere.00549-23-s0001.tif Validation of gene mutant strains.

Figure S2 msphere.00549-23-s0002.tif Effects of defected TrpA, TrpB, and TrpC on mycelial pellet morphology and biomass.

Figure S3 msphere.00549-23-s0003.tif Effect of TrpC on transient response of cytosolic calcium concentration.

Figure S4 msphere.00549-23-s0004.tif Effect of TrpB on the distribution of chitin synthetase ChsB at high temperature.

Figure S5 msphere.00549-23-s0005.tif Sensitivity of mutants to cell wall stress reagents.

Figure S6 msphere.00549-23-s0006.tif Combined effects of TRP mutants and known calcium channels on colony morphology.

Supplemental materials msphere.00549-23-s0007.docx Legends and Tables S1 to S3.

ASM does not own the copyrights to Supplemental Material that may be linked to, or accessed through, an article. The authors have granted ASM a non-exclusive, world-wide license to publish the Supplemental Material files. Please contact the corresponding author directly for reuse.
