# Supplementary figures and images for: The versatility of the putative transient receptor potential ion channels in regulating the calcium signaling in Aspergillus nidulans
Source: mSphere. 2023 Nov 16;8(6):e00549-23. doi: 10.1128/msphere.00549-23 (PMC10732042; doi:10.1128/msphere.00549-23)

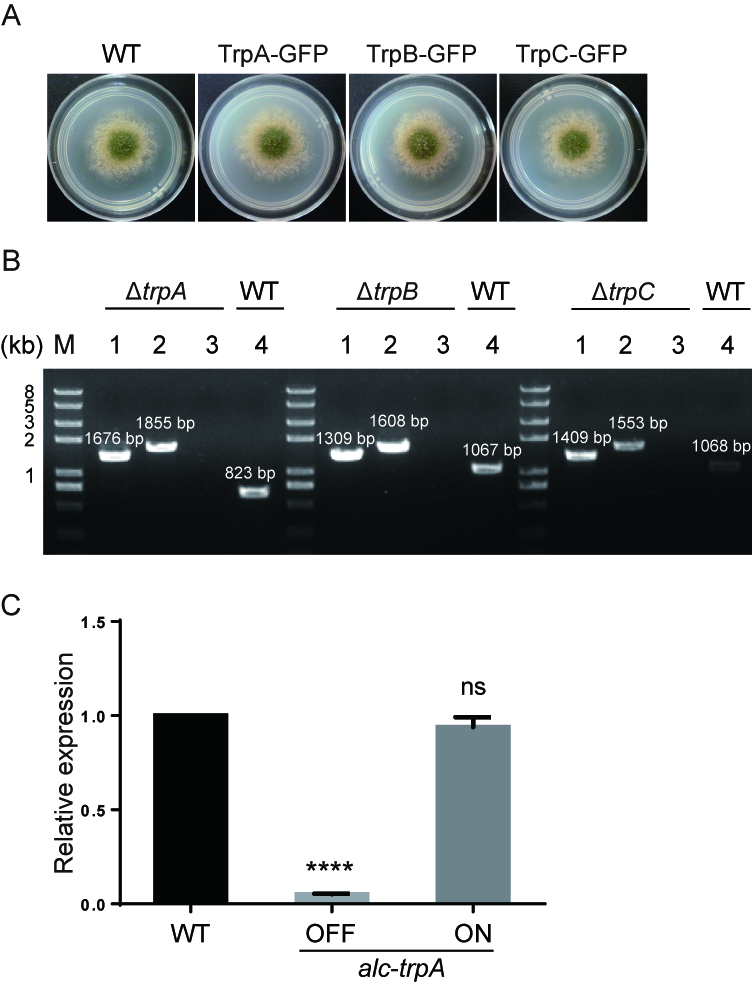

Supplement: Figure S1 — Validation of gene mutant strains. [file msphere.00549-23-s0001.tif]

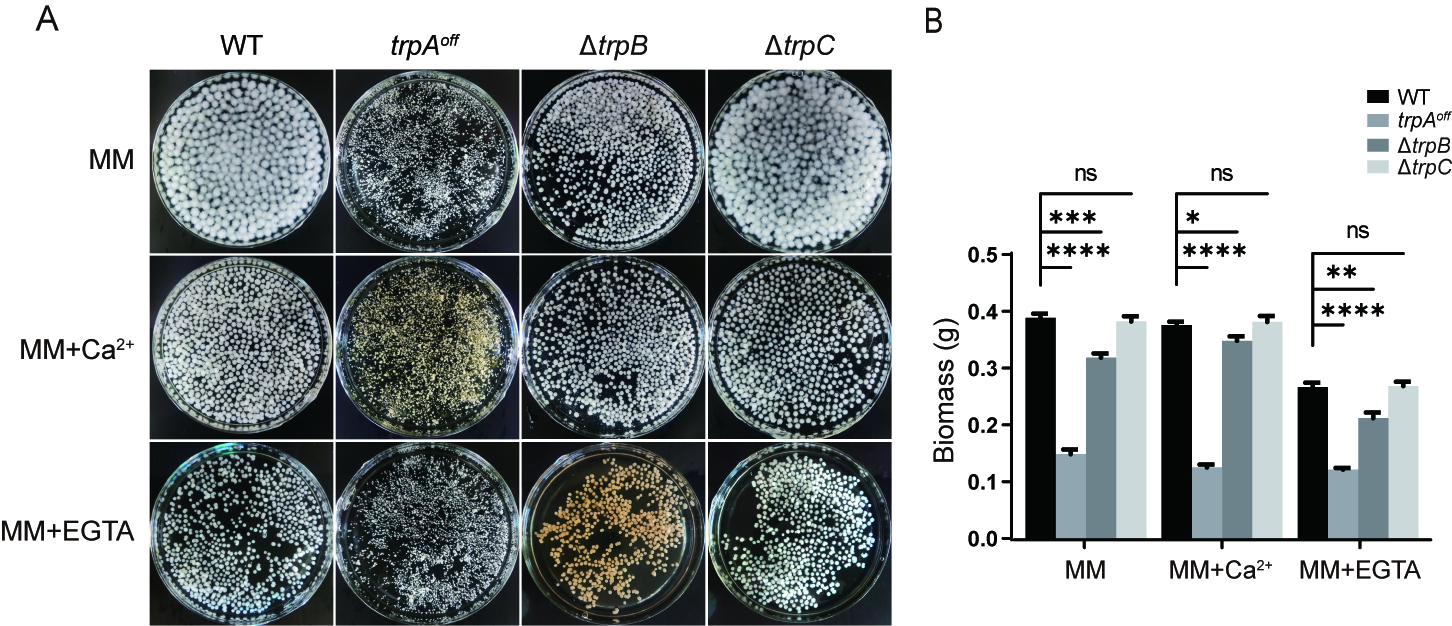

Supplement: Figure S2 — Effects of defected TrpA, TrpB, and TrpC on mycelial pellet morphology and biomass. [file msphere.00549-23-s0002.tif]

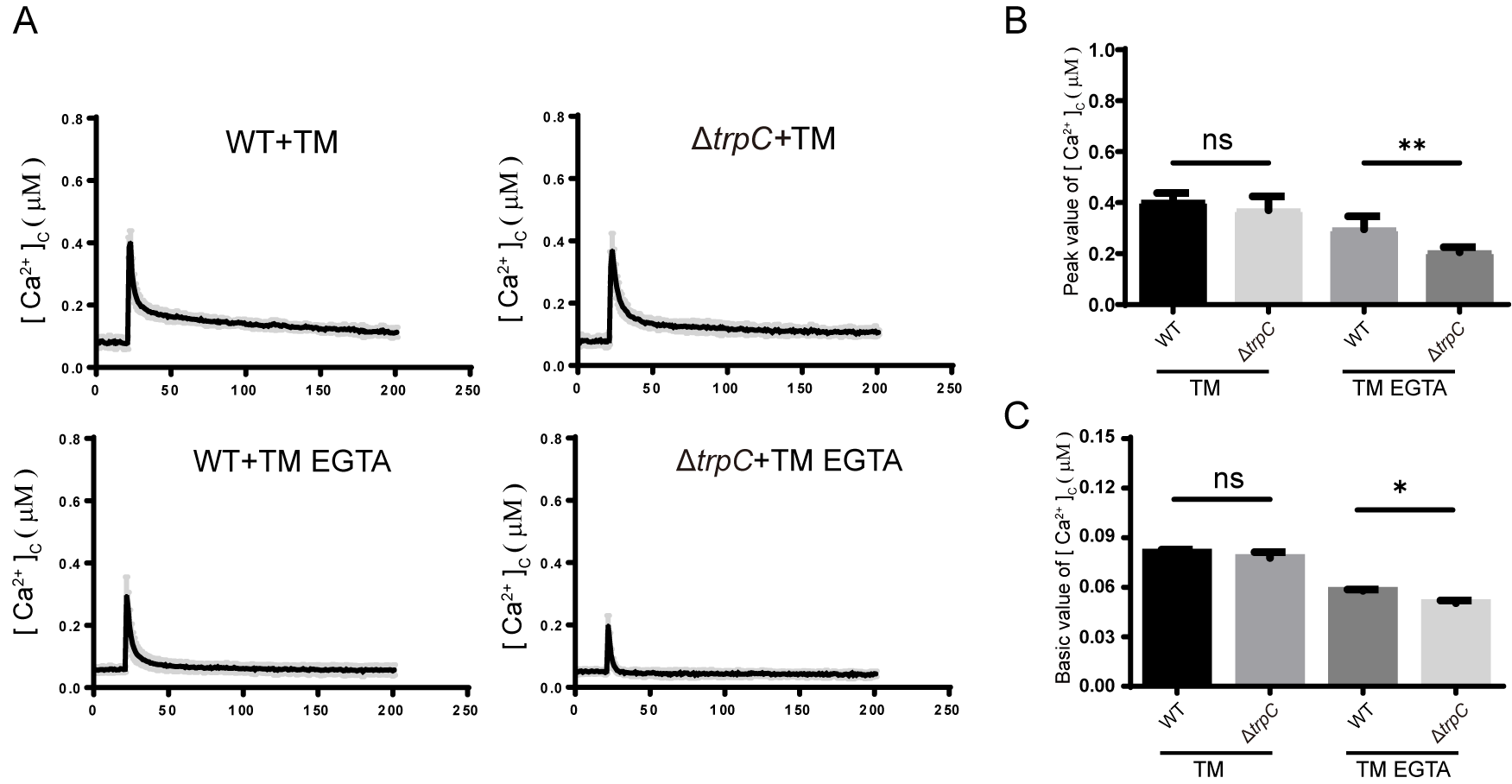

Supplement: Figure S3 — Effect of TrpC on transient response of cytosolic calcium concentration. [file msphere.00549-23-s0003.tif]

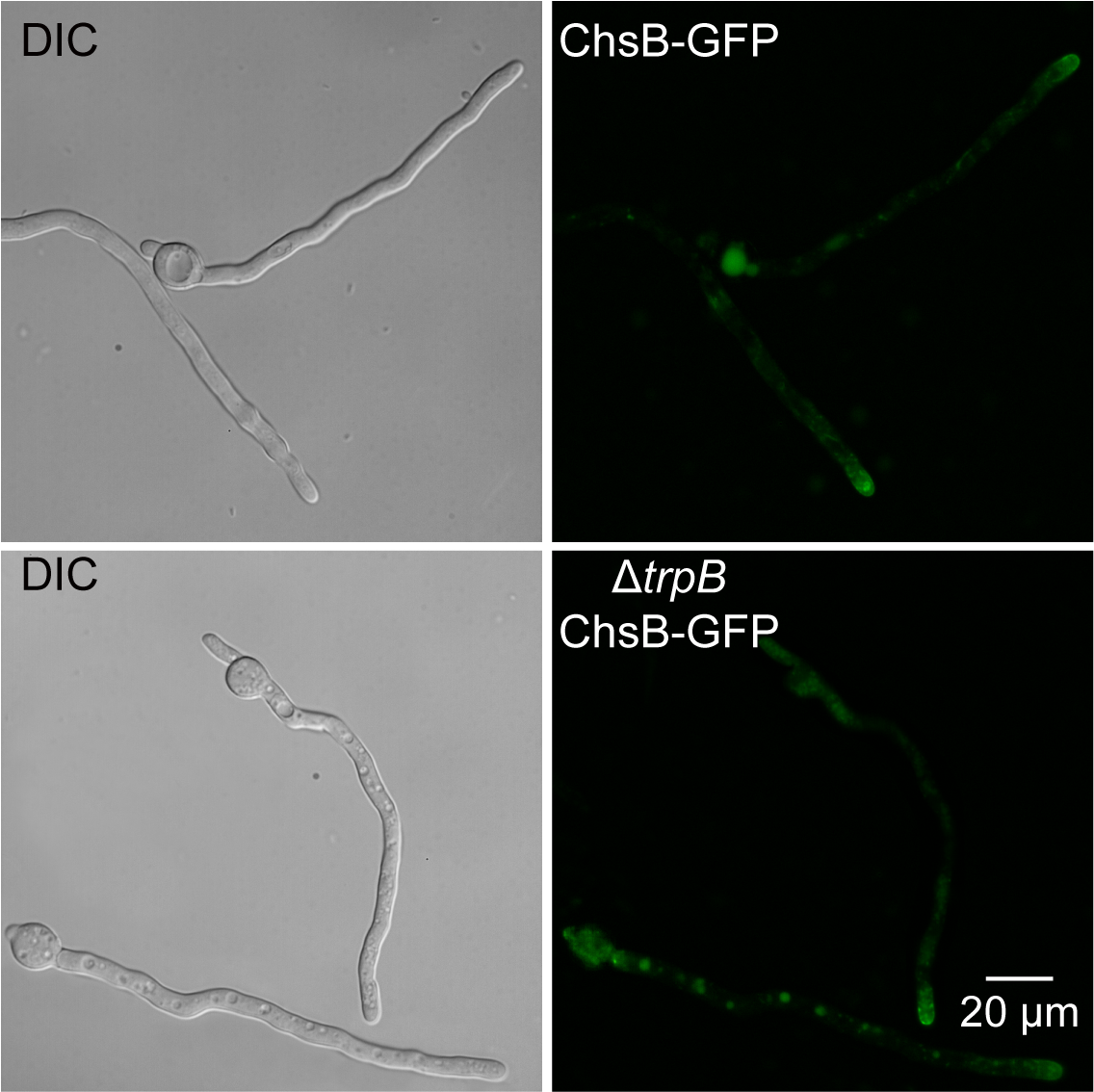

Supplement: Figure S4 — Effect of TrpB on the distribution of chitin synthetase ChsB at high temperature. [file msphere.00549-23-s0004.tif]

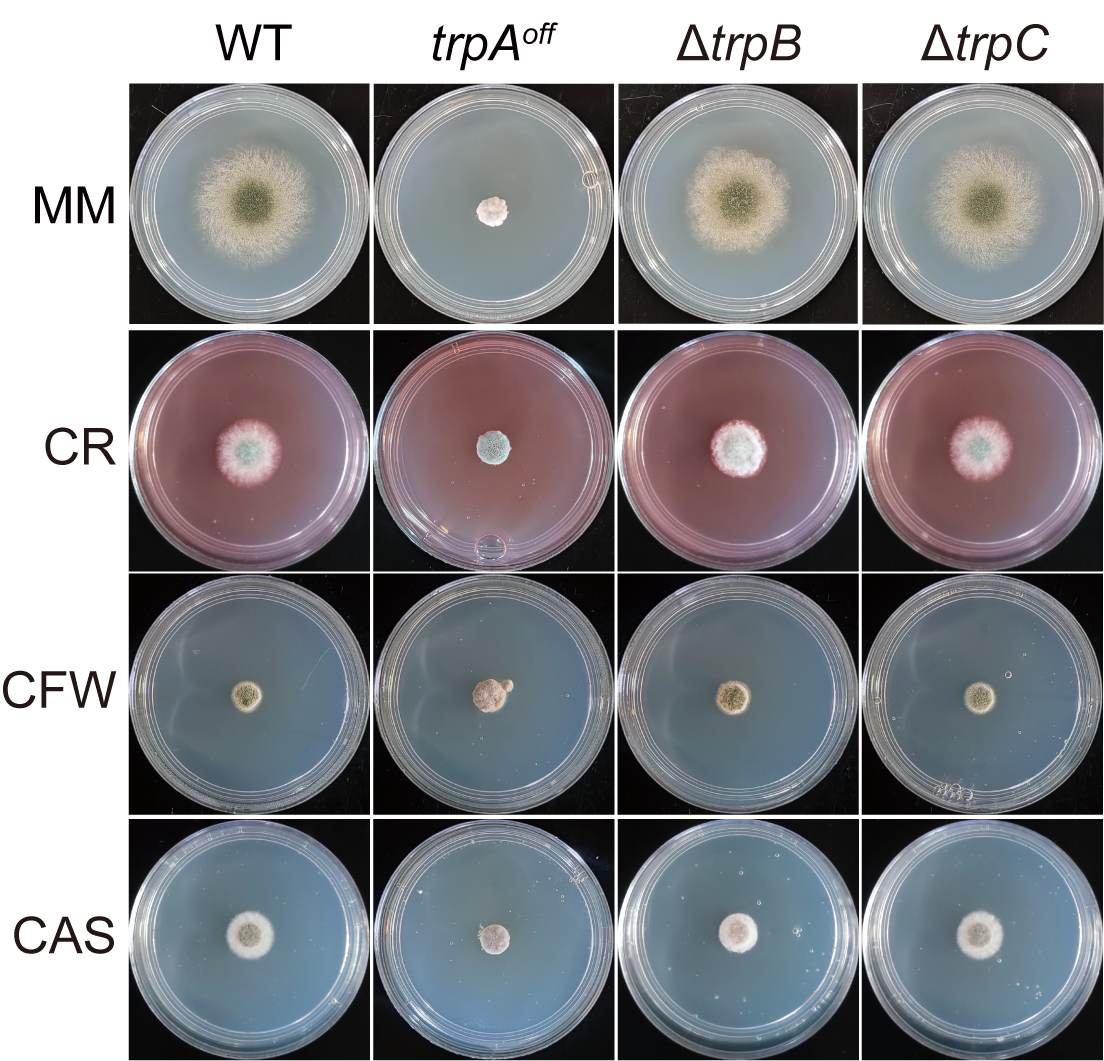

Supplement: Figure S5 — Sensitivity of mutants to cell wall stress reagents. [file msphere.00549-23-s0005.tif]

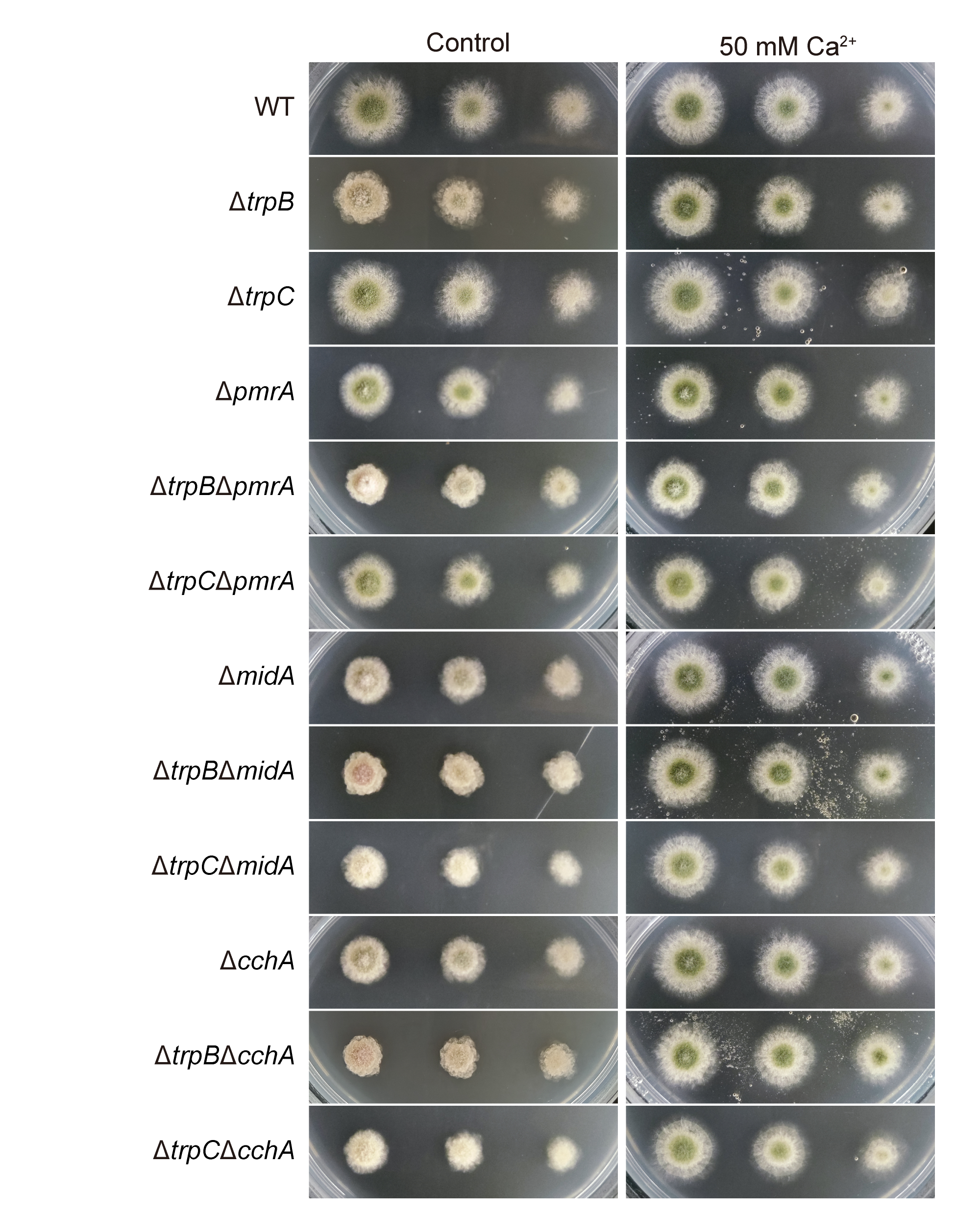

Supplement: Figure S6 — Combined effects of TRP mutants and known calcium channels on colony morphology. [file msphere.00549-23-s0006.tif]
