# Supplementary material for: The versatility of the putative transient receptor potential ion channels in regulating the calcium signaling in Aspergillus nidulans
Source: mSphere. 2023 Nov 16;8(6):e00549-23. doi: 10.1128/msphere.00549-23 (PMC10732042; doi:10.1128/msphere.00549-23)
Supplement: Supplemental materials — Legends and Tables S1 to S3. [file msphere.00549-23-s0007.docx]

**Figure S1.** Validation of gene mutant strains. (A) Validation of colony morphology for the GFP-tagged strains. (B) Diagnostic PCR analyses were performed on the indicated strains, line 1 and line 2, using primer pairs P1/pyrG R and pyrG F/P6, respectively. Line 3 was performed using the primer pair diag F/diag R. Line 4 showed the PCR products with the primer pair diag F/diag R using wild-type genomic DNA as a template. (C) Real-time PCR confirmed the expression level of the *alc-trpA* strain in the off state on glucose medium and in the on state on glycerol expression medium.

**Figure S2.** Effects of defected TrpA, TrpB, and TrpC on mycelial pellet morphology and biomass. (A) Characterizations of submerged fermentation were conducted for the indicated strains using the liquid MMPDRUU medium treated with CaCl_2_ (40 mM) and EGTA (4 mM) at 37 ℃ for 24 h. (B) Biomass analysis was performed for the indicated strains in panel A under all conditions. Values represent mean ± SD from three replicates (ns, not significant; *, P < 0.05; **, P < 0.001; ***, P < 0.001; ****, P < 0.0001).

**Figure S3.** ​Effect of TrpC on transient response of cytosolic calcium concentration. (A) Real-time monitoring of the [Ca^2+^]_c_ of the indicated strains following stimulation with 5 μM tunicamycin (TM) or 5 μM TM supplemented with 0.5 mM EGTA. (B), (C) Quantify the peak and baseline of transient [Ca^2+^]_c_ for the indicated strains shown in Panel C. Values represent mean ± SD from three replicates (ns, not significant; *, P < 0.05; **, P < 0.01).

**Figure S4.** Effect of TrpB on the distribution of chitin synthetase ChsB at high temperature. Epifluorescence observations compare the localizations of the chitin synthetase ChsB in the wild-type strain and the Δ*trpB* mutant. The hyphae were cultured at 42 ℃ for 10 h. DIC microscope image of relative labelled strains on the left. Scale bar: 20 μm.

**Figure S5.** Sensitivity of the *trpA^off^*, Δ*trpB*, and Δ*trpC* mutants to cell wall stress reagents. Colony morphology for the indicated strains grown on MMPDRUU supplemented with 5 mM CR, 20 mM CFW, or 0.1 μM CAS, at 37 °C for 2 days.

**Figure S6.** Combined effects of TRP mutants and known calcium channels on colony morphology. Colony phenotypes of the indicated strains grown on MMPDRUU in the absence and presence of 50 mM CaCl_2_ at 37 °C for 2 days. A series of 2 μL of the 10-fold dilutions were inoculated, which were extracted from a starting suspension of 10^6^ conidia·mL^−1^.

**Table S1.** All *A. nidulans* strains used in this study.

| **Strains** | **Genotype** | **Source** |
| --- | --- | --- |
| TN02A7 | *pyrG89; pyroA4; nkuA::argB2; riboB2; veA1* | FGSC |
| TrpA-GFP | *pyrG89; pyroA4; nkuA::argB2; trpA::GFP::pyrG; riboB2; veA1* | This study |
| TrpB-GFP | *pyrG89; pyroA4; nkuA::argB2; trpB::GFP::pyrG; riboB2; veA1* | This study |
| TrpC-GFP | *pyrG89; pyroA4; nkuA::argB2; trpC::GFP::pyrG; riboB2; veA1* | This study |
| TrpB-GFP  mRFP-PH^OSBP^ | *pyrG89; pyroA4; nkuA::argB2; trpB::GFP::pyrG; riboB2; veA1;*  *gpd::mRFP::PH^OSBP^* | This study |
| Δ*trpA::pyrG* | *pyrG89; pyroA4; nkuA::argB2;* Δ*trpA::pyrG; riboB2; veA1* | This study |
| Δ*trpB::pyroA* | *pyrG89; pyroA4; nkuA::argB2;* Δ*trp**B::pyroA; riboB2; veA1* | This study |
| Δ*trpC::pyroA* | *pyrG89; pyroA4; nkuA::argB2;* Δ*trpC::pyroA; riboB2; veA1* | This study |
| *alc-trpA* | *pyrG89; pyroA4; nkuA::argB2;* Δ*trpB::pyrG; riboB2; veA1* | This study |
| *trpA^c^* | *pyrG89; pyroA4; nkuA::argB2;* Δ*trpA::pyrG; riboB2; veA1; trpA-pyroA* | This study |
| *trpB^c^* | *pyrG89; pyroA4; nkuA::argB2;* Δ*trpB::pyroA; riboB2; veA1; trpB-pyrG* | This study |
| *trpC^c^* | *pyrG89; pyroA4; nkuA::argB2;* Δ*trpC::pyroA; riboB2; veA1; trpC-pyrG* | This study |
| TN02A7-AEQ | *pyrG89; pyroA4; nkuA::argB2; veA1;* pAEQ | This study |
| *alc-trpA*-AEQ | *pyrG89; pyroA4; alc-trpA::pyrG; nkuA::argB2; veA1;* pAEQ | This study |
| Δ*trpB*-AEQ | *pyrG89; pyroA4;* Δ*trpB::pyroA; nkuA::argB2; veA1;* pAEQ | This study |
| Δ*trpC*-AEQ | *pyrG89; pyroA4;* Δ*trpC::pyroA; nkuA::argB2; veA1;* pAEQ | This study |
| Δ*cchA* | *pyrG89; pyroA4; nkuA::argB2;* Δ*cchA::pyrG; riboB2; veA1* | Our lab |
| Δ*midA* | *pyrG89; pyroA4; nkuA::argB2;* Δ*midA::pyrG; riboB2; veA1* | Our lab |
| Δ*pmrA* | *pyrG89; pyroA4; nkuA::argB2;* Δ*pmrA::pyrG; riboB2; veA1* | Our lab |
| Δ*trpB*Δ*cchA* | *pyrG89; pyroA4; nkuA::argB2;* Δ*trpB::pyroA;* Δ*cchA:: pyrG; riboB2; veA1* | This study |
| Δ*trpB*Δ*midA* | *pyrG89; pyroA4; nkuA::argB2;* Δ*trpB::pyroA;* Δ*midA::pyrG; riboB2; veA1* | This study |
| Δ*trpB*Δ*pmrA* | *pyrG89; pyroA4; nkuA::argB2;* Δ*trpB::pyroA;* Δ*pmrA::pyrG; riboB2; veA1* | This study |
| Δ*trpC*Δ*cchA* | *pyrG89; pyroA4; nkuA::argB2;* Δ*trpC::pyroA;* Δ*cchA:: pyrG; riboB2; veA1* | This study |
| Δ*trpC*Δ*midA* | *pyrG89; pyroA4; nkuA::argB2;* Δ*trpC::pyroA;* Δ*midA::pyrG; riboB2; veA1* | This study |
| Δ*trpC*Δ*pmrA* | *pyrG89; pyroA4; nkuA::argB2;* Δ*trpC::pyroA;* Δ*pmrA::pyrG; riboB2; veA1* | This study |

**Table S2.** Recipes for the solutions used in this study.

| **Solution Names** | **Reagent Names** | **Content (mM)** | **Descry** |
| --- | --- | --- | --- |
| MMPDR | glucose | 110 | pH 6.5 |
|  | 1,000 × trace elements | 1 (mL) |  |
|  | 20 × salt solution | 50 (mL) |  |
|  | pyridoxine | 3 (μM） |  |
|  | riboflavin | 15 (μM) |  |
|  | Agar (for solid) | 20 g |  |
| MMPDRUU | uridine | 5 | Based on MMPDR |
|  | uracil | 10 |  |
| 1,000 × Trace element | ZnSO_4_·7H_2_O | 77 | Reagents were added sequentially and the solution was stored at 4 ℃. |
|  | H_3_BO_3_ | 177 |  |
|  | MnCl_2_·4H_2_O | 31 |  |
|  | FeSO_4_·7H_2_O | 18 |  |
|  | CoCl_2_·5H_2_O | 6.7 |  |
|  | CuSO_4_·5H_2_O | 10 |  |
|  | (NH_4_)_6_Mo_7_O_24_·4H_2_O | 1 |  |
| 20 × Salt solution | NaNO_3_ | 1.4 | Solution was stored at room temperature. |
|  | KCl | 0.14 |  |
|  | MgSO_4_·7H_2_O | 0.22 |  |
|  | KH_2_PO_4_ | 0.042 |  |
| PGM | glucose | 50 | pH 6.7 |
|  | MgCl_2_ | 1 |  |
|  | PIPES | 30 |  |

**Table S3.** Primers used in this study.

| **Primer names** | **Sequence 5’ to 3’** |
| --- | --- |
| *trpA* P1 | CTCTGGTCAATGTTAAAGGAT |
| *trpA* P3 | GTAACGCCAGGGTTTTCCTTTGGCGAGTATATGTCCTAC |
| *trpA* P4 | CTATGCGGCATCAGAGCAAAGAGTTTCGCGCGTTTTTTC |
| *trpA* P6 | TGTGGTTTAGGAGCAGGAGAG |
| *trpA* P2 | AGGAGGAAGAGTTCATCATCT |
| *trpA* P5 | GCAGTAATCGCCTGTATATCA |
| *trpA* diag F | TTGGCGACGATGTAGCATCTC |
| *trpA* diag R | GCTGATGACCTTGAATGAAG |
| *trpB* P1 | TCGAAGGCTTCCAACATTCG |
| *trpB* P3 | TGAAGAGCATTGTTTGAGGCTTTGACAAGCACGGGTTGTA |
| *trpB* P4 | CTTGGCATCACGCATCAGTATTCTAGAGTCATAAGTCT |
| *trpB* P6 | AGCTGCACGTTGTCTCTCTA |
| *trpB* P2 | ACCACTTGACCCTTCAAGTC |
| *trpB* P5 | CACACAGCATATGCTGACG |
| *trpB* diag F | GATGACGCCAATTACGTTGAAC |
| *trpB* diag R | TACTTGCGAGCTAGATGTACG |
| *trpC* P1 | TGATCAAGAACTCTTCGGTGG |
| *trpC* P3 | AGGGTGAAGAGCATTGTTTGAGGCTGTTGACCTTTCAAGCCGCT |
| *trpC* P4 | CTTGGCATCACGCATCAGTCATGCCCATCCATTACTTAG |
| *trpC* P6 | GCTTGTTCATCAGGCATGTGA |
| *trpC* P2 | TACTCCCTCTGCCTTGCTGTT |
| *trpC* P5 | CGTTTCAGACAGGCTTGAGA |
| *trpC* diag F | CCTGTATGAGGGCTCATATC |
| *trpC* diag R | CGACCTCGACGAGAAATACGA |
| *alc-trpA* P1 | AGTGGACAACTTGATAAGAGG |
| *alc-trpA* P3 | GTGAAGAGCATTGTTTGAGGCCTGTCGTAGCAACGAAGAG |
| *alc-trpA* P4 | ACTATACAAATAAACCGGTCATGTGGTCAATATCGGTTTTCC |
| *alc-trpA* P6 | GAGCATTATCACAGCGAGGACT |
| *alc-trpA* P2 | TCAGAGGCTGAGCCAGTGGAT |
| *alc-trpA* P5 | CTTCATCTTCCCATGCTTAGC |
| *alc-trpA* diag F | TAGCATTCCGACCCTTCCC |
| *alc-trpA* diag R | GCCTTTCCCTTTATCCTTTG |
| *pyrG* diag F | AGAGTATGCGGCAAGTC |
| *pyrG* diag R | AAACCAGAAGAAACCTCCC |
| *pyroA* diag F | AGAGTATGCGGCAAGTC |
| *pyroA* diag R | AAACCAGAAGAAACCTCCC |
| *trpA^c^* up | AGTGGACAACTTGATAAGAGG |
| *trpA^c^* down | CTATTATCTGACTTACCCGCCAAAGAACGCCGAGTATACGTAG |
| *trpB^c^* up | CAACATGCGATCGGCCAGCAG |
| *trpB^c^* down | CTATTATCTGACTTACCCGCCAATGAAGATTCTGACACTGGAGC |
| *trpC^c^* up | AATCAGGGATTTTGTATCA |
| *trpC^c^* down | CTATTATCTGACTTACCCGCCAAGCTCTCTGGGTGTATGAACT |
| TrpA-GFP P1 | GGCATTGATCAAACGATCTGG |
| TrpA-GFP P3 | TCCAGCGCCTGCACCAGCTCCATGGTCATAACCTGCCCCTC |
| TrpA-GFP P4 | CATCAGTGCCTCCTCTCAGACAGAAGAGTTTCGCGCGTTTTTTCTTC |
| TrpA-GFP P6 | GCTCTCATGTTTTCGCGGTCG |
| TrpA-GFP P2 | GCTAAGCATGGGAAGATGAAG |
| TrpA-GFP P5 | TGGCATGTCAGGCGACATAC |
| TrpB-GFP P1 | GTGTTAGCTCATCATCTGGC |
| TrpB-GFP P3 | TCCAGCGCCTGCACCAGCTCCATAGGCAAACCCATATCCAGGC |
| TrpB-GFP P4 | CATCAGTGCCTCCTCTCAGACAGATTCTAGAGTCATAAGTCTTG |
| TrpB-GFP P6 | AGCTGCACGTTGTCTCTCTA |
| TrpB-GFP P2 | GATACGTCGAGCGGCATAGA |
| TrpB-GFP P5 | CACACAGCATATGCTGACG |
| TrpC-GFP P1 | CTCGTGCTCGACTATTTCCT |
| TrpC-GFP P3 | TCCAGCGCCTGCACCAGCTCCAGCATCGGCCCTCGGCGCCAT |
| TrpC-GFP P4 | CATCAGTGCCTCCTCTCAGACAGCATGCCCATCCATTACTTAGG |
| TrpC-GFP P6 | GCTTGTTCATCAGGCATGTGA |
| TrpC-GFP P2 | ATCCTCGCAGGTGTACTCATAG |
| TrpC-GFP P5 | CGTTTCAGACAGGCTTGAGA |
| PH^OSBP^ F | GCATGCGGAGAGACGGACG |
| PH^OSBP^ R | TCACGAATTCTTCTTCACAGC |
| GFP up | GGAGCTGGTGCAGGCGCTGG |
| *pyrG* down | GCCTCAAACAATGCTCTTCA |
| Aeq F | ATGACCTCCAAGCAGTAC |
| Aeq R | TTAGGGGACGGCACCGCCGTA |
| RT-*tub* F | TCACCTGCTCCGCTATCTT |
| RT-*tub* R | GTTGTTGGGAATCCACTCG |
| RT-*trpA* F | AAGGGCGTTGGATGGG |
| RT-*trpA* R | TGGCGGCAGGTGAATAG |
